# Giant Cell Tumor of Tendon Sheath: A Common Benign Entity With a Sore Note

**DOI:** 10.7759/cureus.43819

**Published:** 2023-08-20

**Authors:** Khushdeep K Shergill, Hari J Pillai, Savijot Singh, Raminderjit Singh

**Affiliations:** 1 Pathology, Indian Naval Ship and Hospital Jeevanti, Vasco Da Gamma, IND; 2 General Surgery, Indian Naval Ship and Hospital Jeevanti, Vasco Da Gamma, IND; 3 General Surgery, Maharishi Markandeshwar Medical College and Hospital, Solan, IND; 4 General Medicine, New Cross Hospital, Wolverhampton, GBR

**Keywords:** soft tissue swelling, surgical excision, magnifying loupe, tendon sheath, giant cell tumour

## Abstract

Giant cell tumor of the tendon sheath (GCTTS) is a slow-growing benign lesion that is reported to be the second most common soft tissue tumor of the hand. Etiopathogenesis remains unexplained, and pre-operative diagnosis is lacking in the majority of cases. A high recurrence rate remains a challenge for the surgeons, with incomplete excision being the most consensually accepted reason. A standard operative protocol of using a magnifying loupe/operating microscope for surgery helps in meticulous dissection and thus reduces the incidence of recurrence in GCTTS. We present the case of a 30-year-old female with a slowly growing nodular lesion on her right index finger, reported as GCTTS post-operatively; however, there was no recurrence at 18 months follow-up because of the use of a magnifying loupe during surgery.

## Introduction

A tenosynovial giant cell tumor is grouped with lesions arising from the synovium of joints, bursae, and tendon sheath [1]. The localized form is called giant cell tumor of tendon sheath (GCTSS), which usually presents as a benign, painless, and slow-growing mass, most commonly on the volar aspect of fingers [2]. It is the reported recurrence of 15%-45% in operated cases, which underlines the challenge it holds for the surgeons in spite of it being the second-commonest soft tissue tumor of the hand [1,3]. Though etiopathogenesis remains unclear, so do the reasons for recurrence; however, several studies in recent times point towards the extent of surgical excision as the most important criteria for recurrence [1]. We report a case with all the classic features of GCTSS but no preoperative diagnosis; however, standard surgical practice played a pivotal role in avoiding recurrence.

## Case presentation

**Figure 1:**
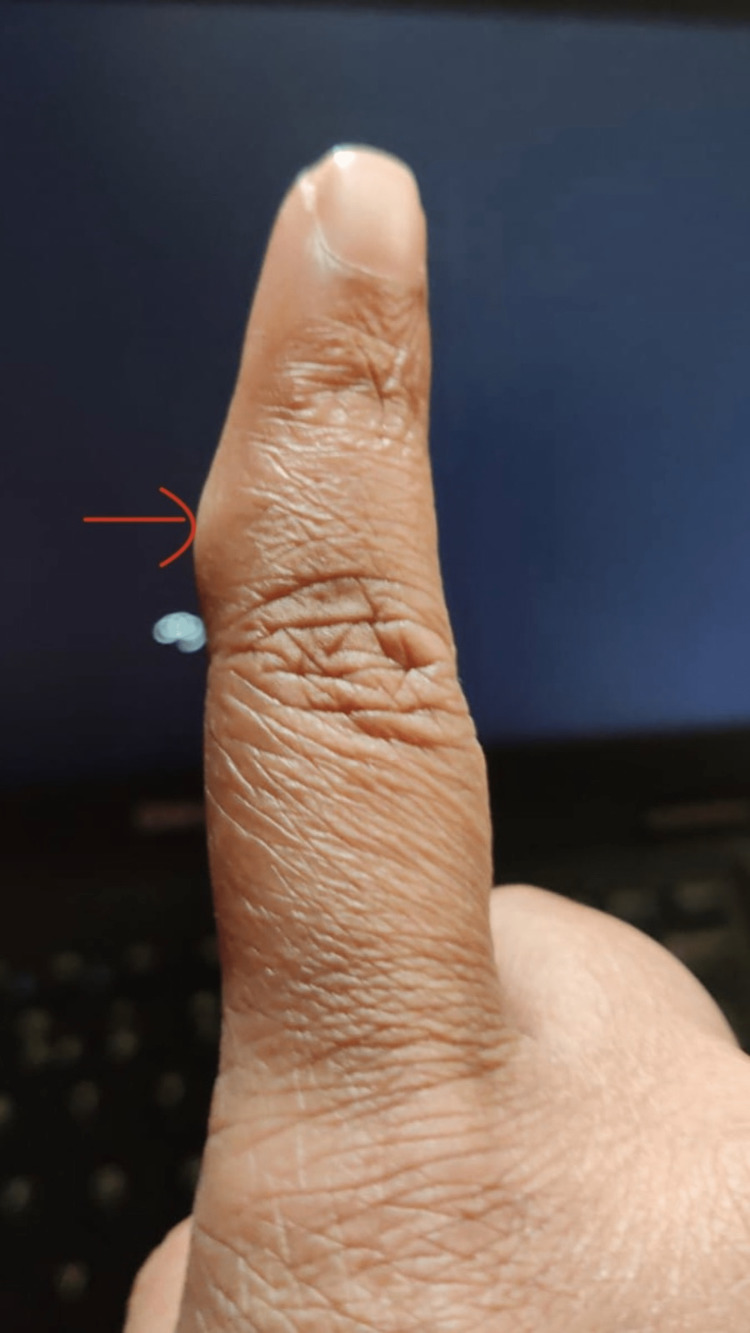
Pre-operative image of a giant cell tumor of the tendon sheath of the right index finger

**Figure 2:**
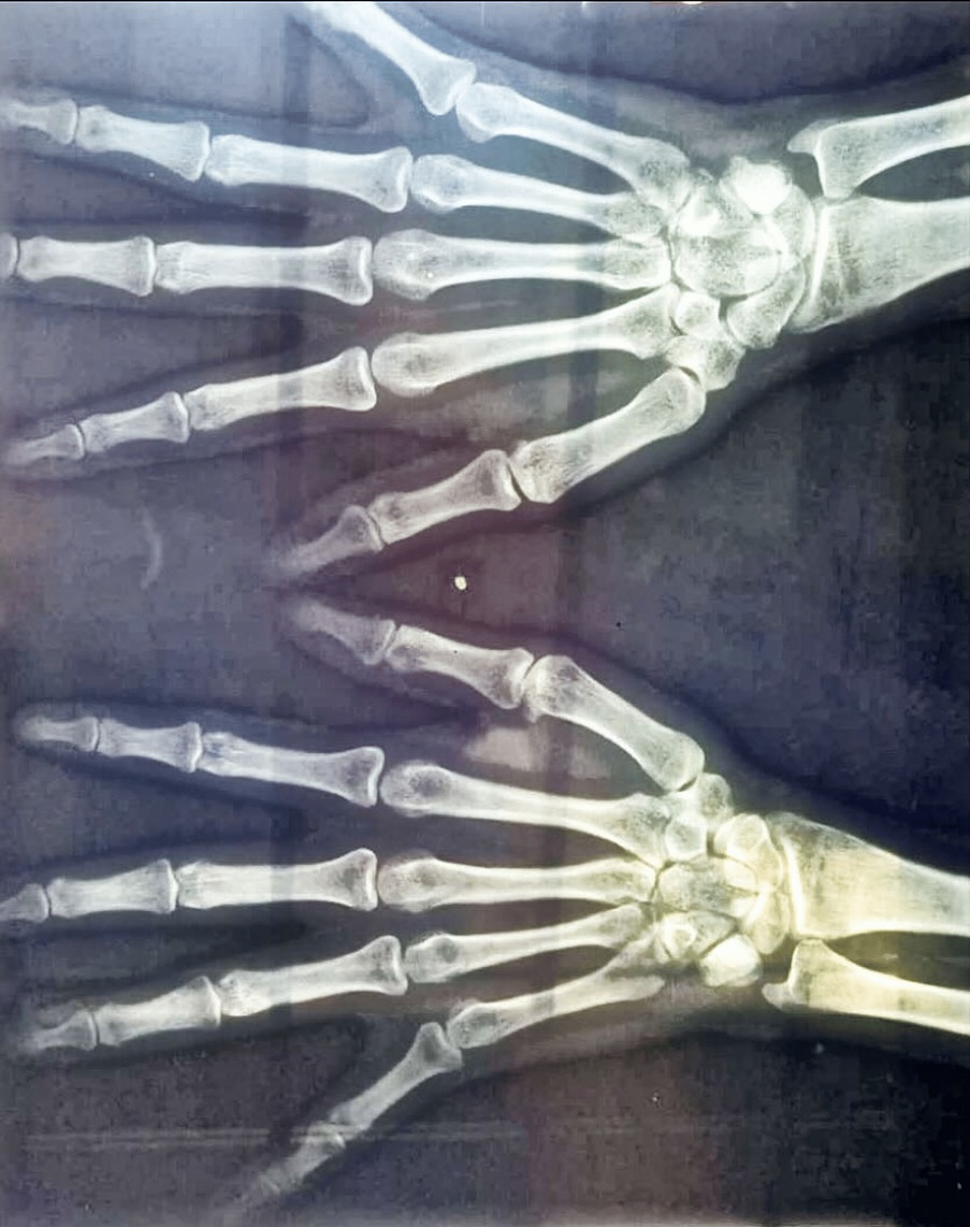
Posteroanterior X-ray of the right hand showing soft tissue swelling and no underlying bony involvement

**Figure 3:**
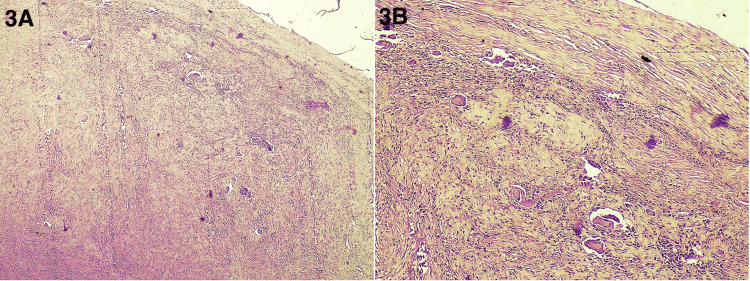
Histopathology (X4 and X10 magnification) shows a well-demarcated lesion comprising osteoclast-like giant cells scattered towards the periphery in a background of mononuclear cells with areas of hyalinization and some hemosiderin deposits

**Figure 4:**
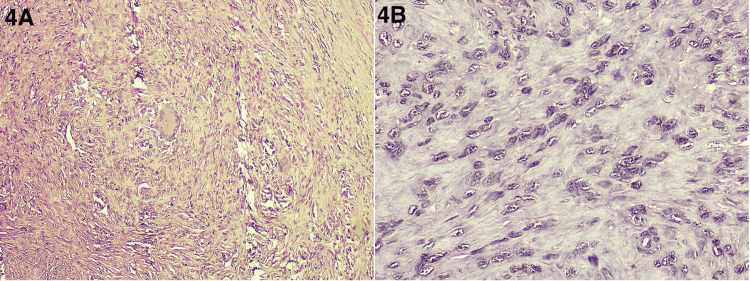
Histopathology (x40 magnification) shows a multinucleate giant cell (4A) and a background of mononucleate cells (4B)

A 30-year-old female patient presented to the surgery OPD with a small nodular lesion on volar aspect of her right index finger (Figure 1). She was a housewife and was concerned if the lesion was cancerous. The lesion progressed from the size of a pinpoint to the present size of 1 x 1 cm over a period of one year. There was no history of repeated trauma, but a history of some trivial injury while chopping coconut was there; therefore, a provisional diagnosis of foreign body granuloma was kept. The lesion was nontender with normal overlying skin. A hand X-ray showed the lesion to be a soft tissue swelling only and ruled out the possibility of any bony tumor (Figure 2). After routine blood investigations, the patient was taken up for surgery with a provisional diagnosis of foreign body granuloma. With an operating microscope of 4.5x magnification, the lesion was excised completely, with no reported satellite lesions or relation to the underlying bone. Histopathological examination confirmed the diagnosis of giant cell tumor of the tendon sheath of the right index finger (Figures 3, 4). The post-operative period was uneventful, and after 18 months of follow-up, there was no recurrence.

## Discussion

Giant cell tumor of the tendon sheath (GCTTS) is a benign, slow-growing, second-most common soft tissue tumor of the hand after ganglion cyst [1]. With a slight female preponderance, it is most seen in the age group of 30-50 years, with the distal phalanx being the most common site of occurrence [1-4]. Several theories regarding its etiopathogenesis have been proposed, ranging from trauma, inflammation, and metabolic disease to a neoplastic etiology with the reported association of chromosomal translocation of 1p13 (CSF1 gene) with these tumors [4]. Based on clinical presentation, these are classified as localized, diffuse, and very rarely malignant. While nodular is the more common type, diffuse is the one seen in larger joints [2,5,6]. In pre-operative workup, X-rays are done to rule out any underlying bony involvement, while USG can differentiate between cystic and solid lesions. MRI, though more helpful in the delineation of lesions, is not confirmatory for diagnosis [1,5,6]. Based on classical history and the site of the lesion, if a diagnosis is suspected, then FNAC showing multinucleate giant cells can help in making a pre-operative diagnosis, which can ensure complete surgical removal, as has been emphasized by Kant K. S. in his review of 26 cases [5,6]. However, histopathology examination is the only confirmatory test because of which diagnosis is made postoperatively in most cases. On biopsy, the tumors show mononuclear cells, which can be small histiocyte-type or large epithelioid cells, osteoclast-like multinucleate giant cells, hemosiderin deposits, areas of fibrosis, and hyalinization. Brisk mitotic activity and necrosis can be seen, while atypical mitosis is associated with malignant forms [4,5]. Complete surgical excision remains the mainstay of treatment. However, despite being a benign tumor, the reported recurrence rate of 15% to 45% still remains a sore point for surgeons and patients alike. In recent times, several studies have been undertaken, such as by Oban et al., Grazia SD et al., and Kant KS et al., to evaluate various factors responsible for recurrence, and as per these, complete surgical excision has been reported as the single most important factor, with emphasis on the importance of magnification used during surgery, which has helped to bring down the recurrence rate to as low as 6% in their studies [1,4,6]. In three cases out of 64 showing recurrence in the study by Grazia SD et al. and two cases showing recurrence in the study by Kant KS et al., the authors reported the primary reason to be incomplete excision of the tumor due to the difficult relationship with surrounding neurovascular structures and the layout of soft tissues [4,6]. Further, the study conducted on 50 operated cases of GCTTS by Ozben et al. also concluded that the use of a magnifying loupe/operating microscope (X4.5) ensures complete excision and hence much lower chances of recurrence. The same was seen in our case, where the lesion was excised with a pre-operative diagnosis of foreign body granuloma; however, the use of an operating microscope ensured complete surgical excision and no reported recurrence 18 months post-surgery. Kant KS also mentions the use of 'Lazy-S' incision and double incision on both dorsal and molar aspects in cases of large tumors encircling the digit so as to give maximum visualization to the operating surgeon [6]. The role of post-operative radiotherapy in cases of inadequate excision and high mitotic index reported on biopsy remains doubtful, and surgical excision remains the mainstay even in cases of recurrence [1,4,5,6].

## Conclusions

In cases with a classic presentation of GCTTS, knowledge of the entity is accompanied by FNAC, whereas in cases with no pre-operative diagnosis, following a standard operative protocol with the use of a loupe/operating microscope for surgery resulting in meticulous dissection can help bring down the incidence of recurrence in GCTTS.
